# Multipronged Electronic Health Record Analysis of Antidepressant Effectiveness on Depression Remission in Patients With Concurrent Depression and Irritable Bowel Syndrome

**DOI:** 10.7759/cureus.64968

**Published:** 2024-07-20

**Authors:** Ashley Deng, Eduardo D Espiridion

**Affiliations:** 1 Psychiatry, Drexel University College of Medicine, West Reading, USA; 2 Psychiatry, Reading Hospital Tower Health, West Reading, USA

**Keywords:** ibs, depression remission, depression in chronic illness, antidepressant drug, ibs treatment

## Abstract

Background

Patients with irritable bowel syndrome (IBS) often experience chronic abdominal pain and bowel habit changes, with a heightened risk of depression and anxiety compared to the general population.

Methods

Using TriNetX data from 61 U.S. healthcare organizations, we conducted a retrospective study of three electronic health record (EHR) analyses. We used International Classification of Diseases, Tenth Revision (ICD-10) and Anatomical Therapeutic Chemical Classification (ATC) codes to analyze depression remission among IBS patients, comparing those using antidepressants to those who were not and comparing outcomes among different types of medication. Statistical methods included risk difference, risk ratio, hazard ratio, Kaplan-Meier survival analysis, log-rank tests, and Cox hazard ratios

Results

Among 78,673 patients with both depression and IBS, those using antidepressants showed significantly higher rates of depressive remission compared to non-users: risk difference (RD), -0.056; risk ratio (RR), 0.380; and hazard ratio (HR), 0.413. Both atypical antidepressants bupropion and trazodone exhibited greater efficacy in achieving remission compared to selective serotonin reuptake inhibitors (SSRIs), sertraline and escitalopram. For SSRI vs bupropion, RD is -0.041, RR is 0.664, and HR is 0.655. For SSRIs vs trazodone, RD is -0.018 , RR is 0.822, and HR is 0.806. The comparative impact of bupropion versus trazodone on remission remains inconclusive.

Conclusion

Depression presents a significant comorbidity in IBS patients, with atypical antidepressants potentially offering superior efficacy in achieving remission compared to SSRIs. Further research should explore these medications' psychiatric outcomes in this population to better understand their therapeutic benefits beyond gastrointestinal (GI) symptoms.

## Introduction

Irritable bowel syndrome (IBS) is a chronic gastrointestinal disorder characterized by symptomology due to unknown etiology, specifically abdominal pain or discomfort and altered bowel habit. IBS is one of the most common gastrointestinal diseases and affects 11% of the global population [1].

Depression and anxiety are often comorbid with IBS with the prevalence of anxiety and depression symptoms in IBS patients being estimated at 39.1% and 28.8%, respectively [2]. In fact, patients with IBS have three times increased odds of having anxiety or depression compared to their healthy counterparts [2]. Anxiety and depression are often inextricably linked and are highly comorbid diseases, with one study finding 41.6% of individuals with major depression also having anxiety [3].

The human gut microbiota, a collection of microscopic organisms that reside in the intestinal tract, demonstrates widespread effects in the body. The microbiota starts development from birth and continues to develop over a lifetime [4]. It can be influenced by genetics, environment, and lifestyle, leading to a unique microbiome that can change over time. Changes in the microbiome that cause negative outcomes are called dysbiosis. When it was discovered that bacteria in the gut could produce neurotransmitters which are also implicated in neurological diseases, the microbiota-gut-brain axis model was developed [5]. The gut-brain axis is a bidirectional model that represents both the anatomical connection between the enteric and the central nervous system and the metabolic connection. This model implicates the immune, circulatory, and neuroendocrine system and the vagus nerve [5].

Mood disorders such as anxiety and depression are linked to gastrointestinal (GI) dysbiosis through modulation of neurotransmitters like gamma-aminobutyric acid (GABA), serotonin, dopamine, and acetylcholine [6]. This is exemplified by serotonin production, where over 90% of the serotonin in the body is produced in the gut by various bacteria [7]. Disruption to the gut as in IBS can therefore affect mood. Anxiety and depression are also associated with inflammation metabolism that can be modulated by the gut through release of cytokines (interleukin (IL)-10, IL-6, tumor necrosis factor (TNF)-alpha, etc.) and inflammasome activation. Gut dysbiosis can impact the integrity of the intestinal barrier, leading to increased permeability, commonly referred to as "leaky gut." This can result in the translocation of bacteria and bacterial endotoxins into the bloodstream, further promoting systemic inflammation [6] and potentially worsening mood disorder. The gut-brain axis, which involves bidirectional communication between the central nervous system and the enteric nervous system, plays a crucial role in maintaining mental health. Alterations in the gut microbiota can affect this communication pathway, influencing mood and behavior.

The well-established relationship between IBS and depression and anxiety has led to studies that explore the impact of antidepressant therapy on IBS symptoms [8-10] but not on psychiatric outcomes. There are even fewer studies that explore the effects of different types of antidepressants, especially atypical antidepressants on psychiatric symptoms. This study will explore the effects of antidepressant therapy on depressive remission in patients with comorbid IBS and depression.

## Materials and methods

Study design

We conducted a retrospective cohort study featuring three components of electronic health record (EHR) analyses. To begin, we compared depression remission in two different cohorts. The control cohort included patients with IBS and concurrent depression without antidepressant medication. The comparison cohort included patients with IBS, depression, and antidepressant medication. For this study, depression was defined as ‘major depressive disorder, recurrent’ (F33), ‘major depressive disorder, single episode, unspecified’ (F32.9), or ‘depression, unspecified’ (F32.A). We matched the two cohorts on age, sex, ethnicity, and race. Using the compare outcomes tool in the platform, we assessed the cohorts for depression remission. Depression remission was defined as ‘major depressive disorder, single episode, in full remission’ (F32.5), ‘major depressive disorder, single episode, in partial remission’ (F32.4), and ‘major depressive disorder, recurrent, in remission’ (F33.4). We analyzed for odds, risk, and hazard ratio of depressive remission.

Next, we aimed to identify the most prevalent antidepressants among patients with IBS and depression who subsequently achieved remission from depression. To ensure the temporal relationship between depressive remission and concurrent IBS, depression, and antidepressant, the first instance of depressive remission had to occur at least one day after any instance of concurrent IBS with antidepressant use and a diagnosis of depression. This way, patients who did not experience depressive remission were excluded. We assessed for the most prevalent antidepressant prescribed in this population to later compare against each other. To compare, we categorized the most common types of antidepressants and ensured that the first instance of a specific antidepressant was identified only after any instance of IBS and concurrent depression. This approach excluded any patients who had been on antidepressants prior to their IBS diagnosis. Although antidepressants can be prescribed concurrently, to focus on a single drug of interest, we intentionally excluded the other two from that cohort. We established three distinct groups following this method. Given that the platform supports comparisons between only two groups at a time, we analyzed the impact of the drugs on depressive remission by comparing Drug A with Drug B, Drug B with Drug C, and Drug A with Drug C. We matched each analysis on age, sex, ethnicity, and race.

Setting

The study was conducted using data from TriNetX, a multi-national collaborative health network spanning over 113 million patient records in 61 healthcare organization systems in the United States alone. TriNetX's data typically dates back to around 2006, coinciding with the implementation of Epic. The data is de-identified, continuously updated, and provides a variety of data points including labs, diagnoses, medications, procedures, and demographics.

Participants

Data for this study was limited to the U.S., spanning 61 healthcare organizations and 113 million records. The control cohort included patients with IBS and concurrent depression without antidepressant medication. The comparison cohort included patients with IBS, depression, and antidepressant medication.

Variables

For this study, depression was defined as ‘major depressive disorder, recurrent’ (F33), ‘major depressive disorder, single episode, unspecified’ (F32.9), or ‘depression, unspecified’ (F32.A). Depression remission was defined as ‘major depressive disorder, single episode, in full remission’ (F32.5), ‘major depressive disorder, single episode, in partial remission’ (F32.4), and ‘major depressive disorder, recurrent, in remission’ (F33.4).

Data source

The data for this study was obtained from TriNetX. IRB approval was not required. The data was queried using International Classification of Diseases, Tenth Revision (ICD-10) codes and Anatomical Therapeutic Chemical Classification (ATC) because the platform employs these methods for querying the electronic medical record. Natural language processing (NLP) is not yet supported on this platform.

Statistical methods

In the two-comparison cohort component of the study, measures of association and survival were assessed using the TriNetX platform. Risk difference and risk ratio were calculated to compare cohorts. Survival was extrapolated using a Kaplan-Meier survival analysis, followed by a log-rank test and Cox hazard ratio and proportionality test. Survival probability of the observed outcome at the end of the time window was calculated and compared. In this study, survival probability refers to the probability that a patient survives without experiencing depressive remission so a higher proportion would indicate fewer patients of that population experienced remission. The log-rank test was used to assess significance (p<0.05). Hazard ratios were calculated using the Cox Proportional hazards model.

## Results

Prior to analyzing the difference in depression remission outcomes in a cohort of IBS patients with comorbid depression prescribed antidepressants versus those that are not, the cohorts were matched for current age, age at index, sex, ethnicity, and race. During propensity score matching, p-values were analyzed to determine if there were statistically significant differences in each characteristic between the matched groups where p<0.05 is considered a statistically significant difference. Chi-Square test was used for categorical variables, and T-test was used for continuous variables. Standardized differences were calculated after matching where a difference <0.1 is considered a negligible difference between the groups. In cohort 1, patients with IBS and comorbid depression prescribed antidepressants, the mean age was 55.0 years, with 75.2% being female and 71.8% identifying as white. In cohort 2, patients with IBS and comorbid depression not prescribed antidepressants, the mean age was 54.9 years, with 75.8% being female and 72.2% identifying as white. Propensity score matching was done to ensure demographic comparability between cohorts. A number of 78,673 patients were included in each cohort with a majority being white females around the age of 55 (Table 1).

**Table 1:** Characteristics of patients prescribed antidepressant and no antidepressant in IBS patients with comorbid depression after matching cohorts on age, sex, ethnicity, and race (n = 78,673) 1=Cohort with IBS patients with comorbid depression and IBS and no antidepressant (N = 78,673). 2=Cohort with IBS patients with comorbid depression and IBS and antidepressant (N = 78,673). IBS: irritable bowel syndrome.

Cohort	Characteristic	Mean ± SD	Patients	% of Cohort	P-Value	Std diff.
1	Age	55.0 ± 19.5	78,673	100%	0.366	0.005
2	Age	54.9 ± 19.3	78,673	100%	0.366	0.005
1	AI (age at index)	49.8 ± 19.4	78,673	100%	0.610	0.003
2	AI (age at index)	49.8 ± 19.2	78,673	100%	0.610	0.003
1	Female		59,176	75.2%	0.009	0.013
2	Female		59,621	75.8%	0.009	0.013
1	Black or African American		5,829	7.4%	0.985	<0.001
2	Black or African American		5,827	7.4%	0.985	<0.001
1	Male		14,791	18.8%	0.570	0.003
2	Male		14,703	18.7%	0.570	0.003
1	White		56,520	71.8%	0.162	0.007
2	White		56,769	72.2%	0.162	0.007
1	American Indian or Alaska Native		334	0.4%	<0.001	0.020
2	American Indian or Alaska Native		240	0.3%	<0.001	0.020
1	Unknown race		12,512	15.9%	0.545	0.003
2	Unknown race		12,600	16.0%	0.545	0.003
1	Native Hawaiian or Other Pacific Islander		319	0.4%	1.000	<0.001
2	Native Hawaiian or Other Pacific Islander		319	0.4%	1.000	<0.001
1	Unknown gender		4,706	6.0%	<0.001	0.019
2	Unknown gender		4,349	5.5%	<0.001	0.019
1	Hispanic or Latino		4,250	5.4%	0.374	0.004
2	Hispanic or Latino		4,330	5.5%	0.374	0.004
1	Not Hispanic or Latino		53,828	68.4%	0.025	0.011
2	Not Hispanic or Latino		54,240	68.9%	0.025	0.011
1	Other Race		2,127	2.7%	0.012	0.013
2	Other Race		1,968	2.5%	0.012	0.013
1	Asian		1,032	1.3%	0.064	0.009
2	Asian		950	1.2%	0.064	0.009

Considering the comparison analysis of patients with IBS and co-existing depression, risk difference of -0.056 indicates that the risk of depression remission is 5.6% lower in the no antidepressant group compared to the antidepressant group. Similarly, risk ratio of 0.380 indicates that the likelihood of depression remission is lower (38%) in the no antidepressant group relative to the antidepressant group. Therefore, risk of remission is higher in the group prescribed antidepressants. The survival probability in the medicated group is lower (76.40%) versus the non-medicated group (89.01%). A lower survival probability suggests a higher probability of remission rate for the medicated group by the end of the study period. The hazard ratio indicates a lower likelihood of remission at any given time for the not-medicated group (HR = 0.413). The p<0.05 in log-rank test confirms significance of difference in time to depressive remission between the two groups (Table 2).

**Table 2:** Measures of association comparison for depressive remission between antidepressant and no antidepressant in IBS patients with comorbid depression IBS: irritable bowel syndrome.

Cohort	Patients in Cohort	Patients with Outcome	Risk	Survival Probability	Metric	Value	95% CI	z	p-value	Chi-squared	df
IBS + depression - antidepressant	78,673	2,699	0.034	89.01%	Risk difference	-0.056	(-0.058, -0.054)	-45.87			
IBS + depression + antidepressant	78,673	7,095	0.09	76.40%	Risk ratio	0.38	(0.364, 0.397)				
					Hazard ratio	0.413	(0.395, 0.432)		0.22	1.504	1
					Log-rank test				0	1631.77	1

Bupropion, an aminoketone, and trazodone, a serotonin antagonist reuptake inhibitor (SARI), were slightly more prescribed than selective serotonin reuptake inhibitor (SSRIs) sertraline and escitalopram in patients who experienced depressive remission (Table 3).

**Table 3:** Antidepressant prescription listed in order from most to least in patients with concurrent IBS and depression taking antidepressants who later experience depressive remission IBS: irritable bowel syndrome.

Medication	Patients	% of Cohort
Bupropion	9,519	45%
Trazodone	8,811	42%
Sertraline	8,525	40%
Escitalopram	7,163	34%

Based on the most prescribed antidepressants, bupropion, trazodone, and sertraline and escitalopram were identified as the medications of interest. Sertraline and escitalopram were grouped together as SSRIs. When comparing bupropion versus SSRI, the risk of remission is 4.1% lower (-0.041) in the SSRI group compared to the bupropion group and patients taking SSRIs are 33.6% (RR = 0.664) less likely to achieve remission compared to those taking bupropion. A higher proportion of patients in the bupropion group achieved remission (survival probability = 71.59%) versus the SSRI group (75.70%). The log-rank test was significant for difference in time to remission (p-value = 0.000). The hazard ratio of 0.655 suggests that patients on SSRIs are less likely to achieve remission at any given time point compared to those on bupropion (Table 4).

**Table 4:** Measures of association of patients with IBS and depression taking bupropion or SSRI only IBS: irritable bowel syndrome, SSRI: selective serotonin reuptake inhibitor.

Cohort	Patients in Cohort	Patients with Outcome	Risk	Survival Probability	Metric	Value	95% CI	z	p-value	Chi-squared	df
SSRI group	21,097	1,692	0.08	75.70%	Risk difference	-0.041	(-0.046, -0.035)	-13.861	0		
Bupropion group	21,097	2,548	0.121	71.59%	Risk ratio	0.664	(0.626, 0.704)	Not Any (NA)			
					Hazard ratio	0.655	(0.616, 0.696)		0.134	2.25	1
					Log-rank test				0	185.29	1

In this cohort, trazodone may be more effective than SSRIs in achieving remission for the outcome of interest. The risk ratio of 0.822 suggests that patients on SSRIs have an 18% lower risk of achieving remission compared to those on trazodone. The confidence interval does not include 1, indicating that this finding is statistically significant; trazodone patients have a lower survival probability (64.96%) compared to SSRI patients (74.17%), indicating a higher rate of remission in the trazodone group. The hazard ratio of 0.806 suggests that patients on SSRIs have a 19.4% lower likelihood of achieving remission at any given time compared to those on trazodone. The survival analysis results indicate that trazodone is associated with a higher rate and quicker time to remission compared to SSRIs in this cohort (Table 5).

**Table 5:** Measures of association of patients with IBS and depression taking trazodone or SSRI only IBS: irritable bowel syndrome, SSRI: selective serotonin reuptake inhibitor.

Cohort	Patients in Cohort	Patients with Outcome	Risk	Survival Probability	Metric	Value	95% CI	z	p-value	Chi-squared	df
SSRI group	22,537	1,832	0.081	74.17%%	Risk difference	-0.018	(-0.023, 0.012)	-6.546	0		
Trazodone group	22,537	2,230	0.099	64.96%	Risk ratio	0.822	(0.774, 0.871)	Not Any (NA)			
					Hazard ratio	0.806	(0.758, 0.857)		0.823	0.05	1
					Log-rank test				0	47.034	1

Risk of remission is higher in bupropion group by 21.5% (RR = 1.215) compared to trazodone group. A lower survival probability indicates a higher rate of remission. Therefore, the trazodone group (64.34%) has a higher rate of remission compared to the bupropion group (71.03%). There is a significant difference in time to remission between bupropion and trazodone groups (p-value = 0.0000). Patients on bupropion have a 20.6% (HR = 1.206) higher likelihood of remission at any given time compared to those on trazodone (Table 6).

**Table 6:** Measures of association of patients with IBS and depression taking bupropion or trazodone only IBS: irritable bowel syndrome.

Cohort	Patients in Cohort	Patients with Outcome	Risk	Survival Probability	Metric	Value	95% CI	z	p-value	Chi-squared	df
Bupropion group	18,540	2,258	0.122	71.03%	Risk difference	0.022	(0.015, 0.028)	6.595	0		
Trazodone group	18,540	1,859	0.1	64.34%%	Risk ratio	1.215	(1.146, 1.287)	Not Any (NA)			
					Hazard ratio	1.206	(1.134, 1.282)		0.115	2.487	1
					Log-rank test				0	35.791	1

## Discussion

After conducting three EHR analyses of IBS patients, we found that in patients experiencing IBS and depression, patients who did not receive antidepressants had a substantially lower likelihood of experiencing remission from depression compared to those who did receive medication. Across the board, patients who experience IBS and concurrent depression were prescribed a wide range of medications from aminoketones, to SSRIs, to serotonin and norepinephrine reuptake inhibitors (SNRI) and more. The use of tricyclic antidepressants (TCAs) due to their adverse effect profile has become less common, while SSRIs have become the most prescribed type of antidepressant [11]. In side-by-side comparisons, bupropion and trazodone, both atypical antidepressants, appear to have more therapeutic effects on depression than SSRIs. When comparing bupropion and trazodone, it is not clear which medication has a more significant impact on increasing depressive remission. Although the risk and hazard ratio support bupropion as being more effective for remission, the survival probability indicates that trazodone has a higher rate of remission. Although SSRIs are the typical first-line treatment for depression and anxiety, in patients with IBS, atypical antidepressants may be more efficacious in achieving depressive remission.

The reason for improved performance of atypical antidepressants versus SSRIs in depression remission is unclear but may be explained by their mechanism of action, adverse effect profile, and unique benefits. We outline a few factors that may make bupropion and trazodone more effective in IBS populations. SSRIs increase availability of serotonin in the synapse by blocking re-uptake into the presynaptic terminal [12]. SSRIs are associated with GI disturbances, especially when beginning treatment or adjusting dosage. Sertraline especially is associated with higher rates of diarrhea [13]. This may be because 90% of serotonin is produced primarily by enterochromaffin cells in the gut [6]. Additionally, patients taking SSRIs are at risk for developing serotonin syndrome, which occurs when there is too much serotonin in the synapses. Serotonin syndrome presents with neuromuscular, autonomic, and mental status symptoms. Nausea and diarrhea are characterized as mild symptoms of serotonin syndrome [14]. SSRIs may not be most effective for treating depression in IBS patients due to its adverse effect profile.

Bupropion is an aminoketone that functions primarily as a norepinephrine and dopamine reuptake inhibitor (NDRI), enhancing the concentrations of these neurotransmitters in the synaptic cleft, which contributes to its antidepressant and anxiolytic effects [15]. Moreover, research has highlighted bupropion's role in modulating the immune system by demonstrating its ability to reduce levels of pro-inflammatory cytokines, specifically TNF-alpha and interferon-gamma [16]. These cytokines are critical mediators in the inflammatory response and are often found elevated in various inflammatory conditions, including IBS.

Trazodone inhibits multiple neurotransmitters that are commonly associated with arousal including serotonin, norepinephrine, dopamine, acetylcholine, and histamine. As such, trazodone is often used as a sedative due to the inhibition of serotonin reuptake and antagonism of H1 and alpha-1-adrenergic receptors [17]. It can be used to treat insomnia and improve sleep quality. Sleep disturbances are common in depressed patients and improving sleep quality has a positive impact on mental health and contribute to remission [18]. While atypical antidepressants are typically reserved for patients who do not tolerate SSRIs well or for those who do not respond to SSRIs initially, considering atypical antidepressants may be advantageous for patients with IBS due to their distinct benefits and lower risk of gastrointestinal side effects.

Limitations

Due to platform limitations, we could not query anxiety remission due to the lack of an ICD-10 code. Anxiety is assessed via tools like the Hamilton Anxiety Rating Scale, typically noted by providers. The platform lacks natural language processing (NLP) for extracting such data. Lack of NLP means that textual information included in the patient's chart cannot be analyzed, which may contain valuable information like symptomology tracking and severity monitoring. Antidepressant dosage and adherence were not assessed. Only three classifications for side-by-side antidepressant comparisons were feasible in TriNetX. Depressive remission was coded using ICD-10, although symptom improvement could potentially be documented in provider notes. Functional impairment in depression and daily activities was not measured. Other confounders besides demographic factors such as chronic diseases were not accounted for because they had variance across the initial cohort population. Despite the constraints of TriNetX, it allows for unprecedented access to an extensive and diverse date repository. These features collectively enable robust and generalizable research, providing valuable insights into depression remission among IBS patients. Future research should explore more antidepressants for depression remission and assess psychiatric outcomes in IBS patients.

## Conclusions

This study provides support that antidepressant therapy significantly improves depressive remission rates in patients with concurrent IBS and depression. Atypical antidepressants, such as bupropion and trazodone, appear to be more effective than SSRIs in achieving depressive remission. These findings advocate for personalized treatment plans and suggest that atypical antidepressants could play a crucial role in the management of depression in IBS patients.
